# Global burden of amputation among children and adolescents from 1990 to 2021: systematic analysis of the Global Burden of Disease study 2021

**DOI:** 10.3389/fpubh.2025.1589309

**Published:** 2025-06-27

**Authors:** Tao Zhang, Qingsong Chen, Hui Li, Wenyu Du, Boyu Li, Ye Cheng, Li Shi, Jianxiao Li, Yue Zhou, Gongbin Wei, Dingyuan Du, Guangbin Huang

**Affiliations:** ^1^Department of Trauma Surgery, Chongqing Emergency Medical Center, Chongqing University Central Hospital, School of Medicine, Chongqing University, Chongqing, China; ^2^School of Microelectronics and Communication Engineering of Chongqing University, Chongqing University Central Hospital (Chongqing Emergency Medical Center), Chongqing, China

**Keywords:** Global Burden of Disease study, epidemiology, amputation, children and adolescents, sex disparities

## Abstract

**Introduction:**

Amputation among children and adolescents, with its substantial physical, psychological, and economic impacts, remains a significant global health issue.

**Objectives:**

This study uses data from the Global Burden of Disease (GBD) to examine trends in amputation burden among youth aged 0–19 from 1990 to 2021.

**Methods:**

This study utilized the 2021 GBD dataset, focusing on the incidence, prevalence, and years lived with disabilities (YLDs) associated with amputations. We applied pinpoint regression and age-period-cohort models to explore trends across the socio-demographic index (SDI) regions, GBD regions, and sex groups from 1990 to 2021, while also forecasting the burden of amputations up to 2040.

**Results:**

We found a global decline in the age-standardized incidence rate (ASIR), age-standardized prevalence rate (ASPR), and age-standardized YLDs rate (ASYR) for children and adolescents. Specifically, the ASIR decreased from 207.3 per 100,000 people in 1990 to 141.5 per 100,000 in 2021, while the ASPR declined from 2,119.6 to 1,437.8 per 100,000 people. Over the same period, YLDs dropped from 30.7 per 100,000 to 18.3 per 100,000. High-SDI countries experienced the greatest declines, reflecting improvements in healthcare and rehabilitation services. In contrast, low-SDI regions and conflict-affected countries like Afghanistan and Syria saw increments in the incidence and prevalence rates. The highest incidence was observed among males aged 15–19 years, with a relative risk of 1.28 compared with the other age groups. Projections indicate that although global incidence rates may continue declining until 2040, disparities in high-risk areas will persist without targeted interventions.

**Conclusion:**

This study highlights a global decline in the burden of amputations among children and adolescents but reveals significant disparities based on SDI regions, sex, and age. Targeted public health strategies and resource allocation are, needed to address these inequalities, improve trauma care, and enhance rehabilitation services, especially in high-risk areas.

## Introduction

Amputation, which involves the surgical removal of a limb or the extremity of a limb, is usually the last resort in critical situations such as severe trauma, infection or tumor (1). Although amputation has clear medical benefits in controlling infections, inhibiting malignant lesions, and restoring blood circulation (2), the physical, psychological, and social burden brought by amputation is enormous. The direct costs of surgery and postoperative care include surgical expenses, inpatient care, rehabilitation, and prosthetic equipment. In the long run, these costs not only impose a heavy burden on individual households, but also put enormous economic pressure on the healthcare system (3, 4). In addition to physical loss, amputation also has profound impacts on the psychological health and social functioning of patients. For children and adolescents, losing limbs not only represents physical disabilities, but also severe emotional trauma, which can affect self-esteem, emotional development, and future social integration (e.g., not having the possibility of getting some jobs) (5, 6). Experiencing life changing events during critical stages of growth can lead to persistent psychological trauma, increasing the risk of post-traumatic stress disorder, depression, and anxiety (7, 8).

Trauma is the main cause of amputation, especially in children and adolescents. Accidents and catastrophic injuries are one of the main causes of limb loss for this group (9). Common causes include road traffic accidents, falls, and injuries caused by war and conflict (10, 11).

Understanding the epidemiological trends of amputations in children and adolescents is crucial for evaluating the effectiveness of public health interventions, particularly those targeting accidental injuries, infections, and other amputation indications (12). Although previous studies have explored the epidemiological trends of amputations in different regions (1, 13, 14), the specific population of children and adolescents has not been fully studied. The Global Burden of Disease (GBD) is a global collaborative research project that systematically integrates all available data to provide consistent and transparent estimates of disability and mortality burdens for most diseases and injuries (15). This study analyzed the global trend of incidence rate, prevalence and years lived with disabilities (YLD) of people aged 0–19 years from 1990 to 2021, emphasizing the differences related to gender, age, access to care, and insufficient diagnosis and treatment. In addition, it also considers socio-economic, medical, and policy factors, predicting trends for the next 19 years. These insights are crucial for developing improved prevention, treatment, and rehabilitation strategies, ultimately helping to alleviate the global burden on this vulnerable group.

## Materials and methods

### Data source and definitions

To assess the current burden and temporal trends of amputations among children and adolescents, we sourced data from the GBD 2021 study (15), which provides aggregate estimates of incidence, prevalence, and YLDs, stratified by sex and age groups. This analysis included individuals under 20 years of age with complete data on the incidence, prevalence, and YLDs for the 1990–2021 period. Cases were excluded if the recorded values for incidence, prevalence, or YLD were zero, if the cause of amputation was unclear, or if the age data were unspecified.

The final dataset provided estimates for the incidence, prevalence, YLDs, and corresponding ASRs by sex and age across 204 countries. Countries were grouped into 21 GBD regions according to geographic proximity and further categorized into five Socio-demographic Index (SDI) quintiles (16). Data processing and verification were conducted in Microsoft Excel 2021 using a double-entry system to ensure accuracy. YLDs were defined as the aggregate number of years lived with any short-term or long-term health loss due to amputation, adjusted by disability severity weights. Amputations were identified based on codes from the Ninth (ICD-9) and Tenth (ICD-10) revisions of the International Classification of Diseases.

### Descriptive analysis

This study utilized the DisMod-MR 2.1 Bayesian hierarchical model from the GBD 2021 database to estimate the incidence, prevalence, and YLDs attributable to amputations. DisMod-MR 2.1 is a multiparameter, comprehensive regression model specifically developed for disease burden estimation, integrating data from diverse sources (e.g., hospital records, epidemiological surveys, and population questionnaires) to enhance the accuracy and comparability of estimates across regions and populations (17).

The model follows GBD’s standardized methodologies for estimating disease and injury burden, leveraging input data on incidence, prevalence, recovery, recurrence, and mortality rates. DisMod-MR 2.1 uses Bayesian statistical methods to address data uncertainty, producing age- and sex-specific burden estimates for amputations. Results are expressed as the age-standardized incidence rate (ASIR), age-standardized prevalence rate (ASPR), and age-standardized YLDs rate (ASYR), accompanied by 95% uncertainty intervals (UIs) to reflect the estimation precision. All calculations and data analyses were performed within the GBD database system, with double data entry and validation checks implemented to ensure data reliability and consistency.

### Age-period-cohort analysis

This study applied an age-period-cohort model to decompose trends in amputation incidence, prevalence, and YLDs across various age groups, time periods, and birth cohorts. The age-period-cohort model, frequently used in epidemiological studies of noncommunicable diseases, enables the attribution of temporal changes in the amputation burden to distinct age, period, and cohort effects. The baseline group for the age dimension in all analyses was the 0–4 years age group. The age effect captures variations in amputation risk across age groups, accounting for physiological, psychological, and behavioral differences, with older populations often exhibiting higher risks. The period effect reflects the influence of environmental factors, healthcare advancements, and public health policies on the amputation burden over specific periods. The cohort effect examines risk exposures unique to groups of people born in different years, often linked to early-life health status or environmental exposures (18–20).

To address collinearity among age, period, and cohort, this study utilized the Intrinsic Estimator method, enhancing model identifiability and enabling an independent estimation of each effect’s contribution (21). Model calculations were conducted using Stata software (Version 16.0; StataCorp), employing relative risk (RR) to evaluate age-specific risks across periods and cohorts. The significance of the effects was assessed with the Wald chi-square test, with the threshold for statistical significance set at *p* < 0.05. Notably, the APC results are presented for descriptive purposes only.

### Joinpoint regression model

This study employed a Joinpoint regression model to analyze temporal trends in the incidence, prevalence, and YLDs associated with amputations, estimating the annual percent change (APC) and average annual percent change (AAPC) in amputation burden from 1990 to 2021 (22). Joinpoint regression, a segmented linear regression method, detects “joinpoints” within a time series to highlight significant shifts in trends, enabling independent assessments of change rates across distinct periods.

In this analysis, the Joinpoint regression model iteratively added pinpoints to the dataset to identify the optimal segmentation, calculating the APC for each segment to determine both the direction and magnitude of trends in each period. The AAPC provided an average change estimate across the entire study period, reflecting the overall trend trajectory. An APC or AAPC greater than 0 indicated an upward trend in amputation burden, while values less than 0 indicated a downward trend; values equal to 0 suggested no significant trend change (23).

Model fitting and APC/AAPC calculations were performed using Joinpoint (version 4.9.1.0), with all results reported at a 95% CI. The threshold for statistical significance was set at *p* < 0.05 to identify meaningful trend changes. Our Joinpoint analysis used non-constant variance modeling to fully incorporate the UIs in the estimation process.

### Measurement of health inequalities

This study utilized health inequality analysis methods to evaluate disparities in amputation burden across regions with varying SDI levels. To quantify both absolute and relative inequalities, we applied the Slope Index of Inequality (SII) and the CI. The SII regresses age-standardized amputation incidence rates against the income-related social rank of each country, measuring health burden disparities across income groups, with higher SII values indicating greater inequality. Conversely, the CI assesses the distribution of amputation burden across income levels using a Lorenz curve, where a negative CI value signifies a concentration of burden in lower-income groups, and a positive CI value reflects concentration in higher-income groups (24).

In this analysis, countries were first ranked according to gross domestic product (GDP) per capita, and each country’s relative social position was determined based on the cumulative population distribution. The SII was then calculated using a weighted regression model, with adjustments for heteroscedasticity and non-linear effects made by applying log-transformed GDP values. CI calculations were performed using the *egen_inequal* package in Stata (version 16.0), where the numerical integration of the Lorenz curve’s area provided an assessment of inequality in amputation burden.

### Future burden prediction

This study employed the Nordpred forecasting model to project future trends in the burden of amputations. Based on the age-period-cohort framework, the Nordpred model accounts for demographic shifts, evolving disease trends, and generational effects to provide long-term estimates of amputation incidence and YLDs. The prediction analysis spans 2022 to 2040, with the model fitting based on historical data from 1990 to 2021 to identify patterns in future burdens.

The Nordpred model utilizes a log-linear regression approach, implemented via the Nordpred package in R (version 4.3.2) (25, 26). To enhance predictive validity, the model adjusts the slope of the time series based on recent trends, limiting linear extrapolation to more accurately reflect realistic disease progression. Specifically, the model establishes a trend line using historical data and then projects future points according to demographic and epidemiological factors.

The prediction results are presented with 95% CIs to ensure precision. A significance threshold of *p* < 0.05 was applied, with statistical significance assessed using the Wald chi-square test.

### Frontier analysis

To assess the relationship between the burden of amputation in children and adolescents and socio-demographic development, we conducted frontier analysis separately for ASIR, ASPR, and ASYR (27). To this end, we used data from GBD 2021, which includes health burden information for countries and regions worldwide. First, we applied Data Envelopment Analysis (DEA) to construct the frontier for each country or region at a given SDI level, in order to evaluate the minimum potential burden relative to their development level. For ASIR, ASPR, and ASYR, we generated three separate frontier models to analyze the efficiency differences for each indicator globally. During the analysis, we considered the specific SDI value of each country or region and used the Free Disposal Hull (FDH) method to construct non-linear frontier boundaries. Additionally, we applied local polynomial regression (LOESS) to smooth the frontier curves, ensuring the robustness of the analysis results. Through this frontier analysis approach, we were able to quantify the gap between each country or region and the global frontier, reveal potential unrealized gains, and provide targeted recommendations for policy development.

## Results

### 2021 global distribution of amputations among young and adolescent populations

**Figure 1:**
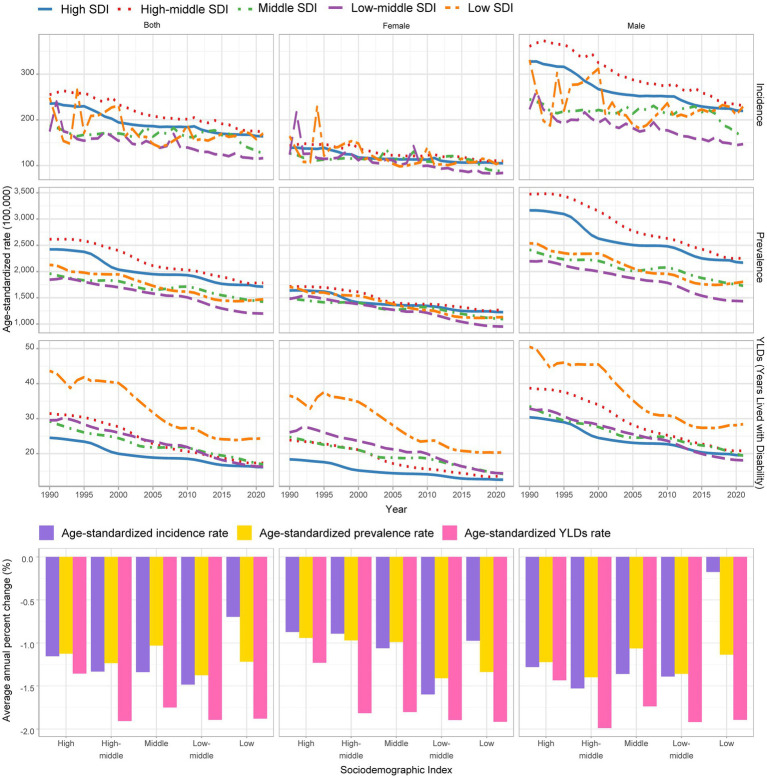
Global trends in ASIR, ASPR, and ASYR for amputations among children and adolescents aged 0–19 across different SDI groups (1990–2021).

Data from 2021 on the global burden of amputation among children and adolescents aged 0–19 years indicate an ASIR, ASPR, and ASYR of 141.532 per 100,000 population (95% UI: 109.676–183.304), 1437.846 (95% UI: 1237.270–1704.587), and 18.3 (95% UI: 11.767–28.754), respectively. When classified by SDI, the data revealed that the burden of amputation was significantly higher in high–middle-SDI countries than in other regions, with ASIR and ASPR values of 173.411 (95% UI: 128.367–233.827) and 1781.688 (95% UI: 1507.117–2149.634). In contrast, countries with low-SDI levels presented similar ASIRs (170.424, 95% UI: 126.484–231.153) but the highest ASYR (24.43, 95% UI: 16.298–35.835), (Figure 1; Supplementary Tables S1–S3). This disparity may be related to the limitations in the healthcare infrastructure and resources in these countries. Although countries with higher SDIs show elevated ASIRs and ASPRs, their lower ASYRs suggest more effective amputation outcomes and rehabilitation programs. Conversely, countries with limited healthcare resources encounter significant challenges in prognosis and rehabilitation, leading to higher ASYRs. Regional analyses highlight the fact that the highest burden of amputations in young and adolescent populations is particularly evident in Australasia (ASIR: 447.946, 95% UI: 292.612–668.914; ASPR: 4741.802, 95% UI: 3746.814–5935.112) and Central Europe (ASIR: 476.11, 95% UI: 321.801–686.138, ASPR: 4611.373, 95% UI: 3770.303–5663.075) (Supplementary Tables S1–S3).

Sex differences are also pronounced. Globally, the ASIR, ASPR, and ASYR for males were 184.965, 1763.642, and 21.07, respectively, which were significantly higher compared with the 95.464, 1092.518, and 15.366 for females (Figure 1; Supplementary Figure S1; Supplementary Table S1). Age also plays a crucial role, with the incidence, prevalence, and YLDs all rising with age, especially in the 15–19 years age group, where the disease burden is most severe among males (Supplementary Figures S2–S4; Supplementary Table S4).

When comparing SDI regions across different age groups, it is evident that the ASIR and ASPR for individuals under 15 years in high–middle-SDI countries are much higher than those in other regions, while ASYR remains elevated across all age groups in low-SDI regions. On the other hand, the incidence in the 15–19 years age group was significantly higher in low-SDI countries than in other nations (Supplementary Figure S2). Across all SDI regions, ASIR, ASPR, and ASYR were consistently higher in males than in females, with these disparities becoming more pronounced with age (Supplementary Figure S5).

### Time trend of amputation among young and adolescent patients from 1990 to 2021

From 1990 to 2021, the incidence, prevalence, and YLDs of amputations among children and adolescents have shown significant global declines across various SDI categories. The global ASIR decreased from 207.291 per 100,000 population in 1990 (95% UI: 160.512–267.711) to 141.532 in 2021 (95% UI: 109.676–183.304), with an AAPC of −1.124 (95% CI: −1.249 to −1). High-SDI countries saw a more rapid decline, with an AAPC of −1.155 (95% CI: −1.313 to −0.995), compared to −0.697 (95% CI: −1.279 to −0.112) in low-SDI countries (Figure 1; Supplementary Table S1).

The global ASPR declined from 2119.564 (95% UI: 1817.503–2502.456) in 1990 to 1437.846 (95% UI: 1237.27–1704.587) in 2021, with an AAPC of −1.266 (95% CI: −1.363 to −1.17). The ASPR decline was slower in high-SDI countries than in low-SDI countries, with an AAPC of −1.124 (95% CI: −1.22 to −1.029) and −1.218 (95% CI: −1.367 to −1.069), respectively (Figure 1; Supplementary Table S2).

The global ASYR fell from 30.706 (95% UI: 20.584–47.042) in 1990 to 18.3 (95% UI: 11.767–28.754) in 2021, showing an AAPC of −1.683 (95% CI: −1.84 to −1.525). The ASYR declined more slowly in the high-SDI regions than in the low-SDI regions, with an AAPC of −1.356 (95% CI: −1.427 to −1.285) and −1.88 (95% CI: −2.144 to −1.616), respectively (Figure 1; Supplementary Table S3).

Regional trends reveal that Eastern Sub-Saharan Africa experienced the most substantial decline in incidence (AAPC: −2.175), prevalence (AAPC: −2.082), and YLDs (AAPC: −3.07) between 1990 and 2021, while the Caribbean region showed an increasing burden (AAPC of ASIR: 0.281; ASPR: 0.571; ASYR: 1.587). In most regions, both male and female populations exhibited similar downward trends, with declines being more pronounced for females than males in Tropical Latin America (Supplementary Figure S6). Detailed AAPC analysis across regions showed that the middle-SDI region experienced the greatest for females than males in decline in incidence from 2016 to 2021, with an APC of −6.39%. The global prevalence dropped substantial between 2010 and 2015, with an APC of −2.30%. YLDs decreased notably during 2000–2004, with an APC of −3.00%, and further declined during 2010–2015, reaching an APC of −2.57% (Supplementary Figure S7).

When separating the population into children (0–9 years) and adolescents (10–19 years) with a cut-off at 9 years, the global incidence AAPC was −1.31% for children and −1.19% for adolescents from 1990 to 2021. The AAPC for ASPR during the same period was −1.48% for young children and −1.19% for adolescents. The AAPC for YLDs was −1.93 and −1.59%, respectively (Supplementary Figures S8, S9). These findings indicate that although the overall burden of amputations is decreasing globally, there are still substantial variations across regions and age groups.

The global map of ASIR, ASPR, and ASYR for 2021, along with the national rates and AAPC of amputations among children and adolescents between 1990 and 2021 were also explored. In 2021, Afghanistan recorded the highest ASIR (1042.14, 95%UI: 622.441–1691.842), while the Democratic People’s Republic of Korea (52.217, 95%UI: 42.245–65.34), Taiwan (Province of China) (53.066, 95%UI: 40.882–70.275), and Kiribati (53.633, 95%UI: 41.172–70.099) had significantly lower ASIRs compared with other countries. Similarly, the Syrian Arab Republic (8263.689, 95%UI: 4961.038–13243.409) exhibited the highest ASPR, whereas Kiribati (526.228, 95%UI: 459.83–624.622), the Democratic People’s Republic of Korea (557.39, 95%UI: 496.294–643.373), and Taiwan (Province of China) (616.258, 95%UI: 538.662–734.392) showed significantly lower ASPRs. Regarding the ASYR, Afghanistan and the Syrian Arab Republic had significantly higher YLDs than other countries, with values of 123.537 (95%UI: 68.425–212.344) and 133.092 (95%UI: 66.147–241.161), respectively. Taiwan (Province of China) (6.174, 95%UI: 3.299–10.917) and the Democratic People’s Republic of Korea (7.74, 95%UI: 4.832–12.183) continued to maintain the lowest ASYRs. The primary reason behind these disparities may be social factors such as conflict and war. From 1990 to 2021, Eritrea experienced the most significant decline in the ASIR, with an AAPC of −10.071 (95%CI: −16.618 to −3.011). In contrast, Yemen (4.11, 95%CI: −0.1–8.497) and Afghanistan (3.864, 95%CI: −0.008–7.885) showed the most pronounced increments. Similarly, Eritrea also had the most significant decrease in the ASPR, with an AAPC of −7.202 (95%CI: −7.956 to −6.442), whereas the Syrian Arab Republic exhibited the most notable increase (4.711, 95%CI: 3.873–5.556). Regarding the ASYR, Eritrea again showed the most substantial decline (−9.678, 95%CI: −10.558 to −8.79), while the Syrian Arab Republic (5.035, 95%CI: 4.015–6.065) and Haiti (2.619, 95%CI: 1.472–3.78) experienced significant increases (Supplementary Figures S10–S12; Supplementary Tables S1–S3).

### Age–period–cohort analysis

**Figure 2:**
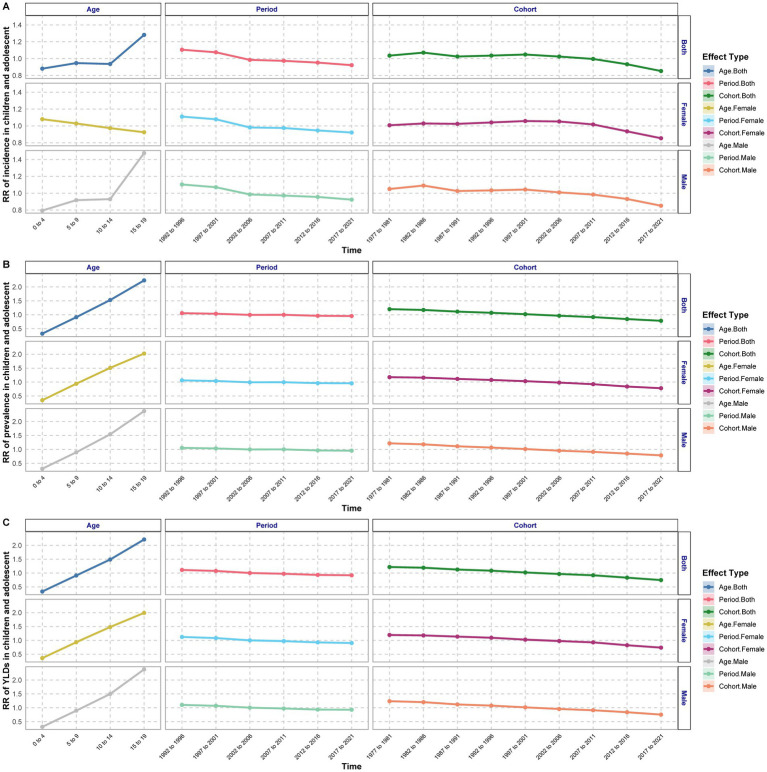
Estimated relative risks of age, period, and cohort effects on the incidence **(A)**, prevalence **(B)**, and YLDs **(C)** of amputations among children and adolescents.

Age effect analyses revealed that ASIR, ASPR, and ASYR of amputation increase with age. In terms of incidence, the lowest risk was observed in the 0–4 age group (RR = 0.881), while the highest risk was seen in the 15–19 age group (RR = 1.281). For males, the incidence initially rose to a plateau before increasing again, with the lowest risk in the 0–4 age group (RR = 0.794) and the highest risk in the 15–19 age group (RR = 1.477). In contrast, the incidence trend for females continued to decline, with the lowest risk in the 15–19 years age group (RR = 0.925) and the highest risk in the 0–4 age group (RR = 1.08). The ASPR risk was lowest in the 0–4 age group (RR = 0.321) and highest in the 15–19 age group (RR = 2.235). The YLD risk was also lowest for the 0–4 age group (RR = 0.333) and highest for the 15–19 age group (RR = 2.212) (Figure 2A; Supplementary Table S4).

The period effect analysis indicated that the ASIR, ASPR, and ASYR of amputation among children and adolescents exhibited a continuous downward trend in the general, male, and female populations. The lowest ASIR rates were recorded from 2017 to 2021 across all groups (general: RR = 0.922; male: RR = 0.924; female: RR = 0.922). The highest ASIR rates were observed between 1992 and 1996 (general: RR = 1.105; male: RR = 1.104; female: RR = 1.111). Similarly, the lowest ASPR values were noted from 2017 to 2021 (general: RR = 0.955; male: RR = 0.954; female: RR = 0.957). The highest ASPR was recorded during 1992–1996 (general: RR = 1.057; male: RR = 1.054; female: RR = 1.061). The lowest YLDs were also observed from 2017 to 2021 (general: RR = 0.92; male: RR = 0.929; female: RR = 0.907). The highest YLD rates occurred between 1992 and 1996 (general: RR = 1.111; male: RR = 1.103; female: RR = 1.123) (Figure 2B; Supplementary Table S4).

Cohort effect analyses showed a fluctuating pattern in the ASIR of amputation among children and adolescents, characterized by increases, decreases, and subsequent variations. The highest overall risk was observed in the 1982–1986 cohort (RR = 1.071), while the lowest risk was found in the 2017–2021 cohort (RR = 0.852). Both ASPR and YLDs exhibited a continuous decline overall. The highest risk was recorded in the 1977–1981 cohort (ASPR: RR = 1.201; YLDs: RR = 1.218), while the lowest overall risk was in the 2017–2021 cohort (ASPR: RR = 0.783; YLDs: RR = 0.746) (Figure 2C; Supplementary Table S4).

### Analysis of the transnational inequality

**Figure 3:**
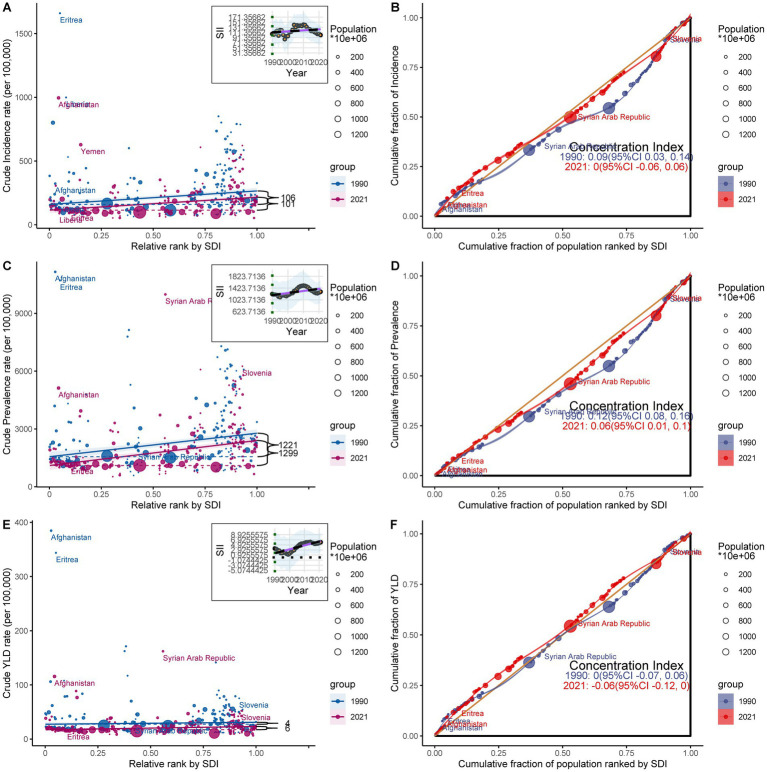
Health inequality regression curves **(A,C,E)** and concentration curves **(B,D,F)** for the incidence, prevalence, and YLDs of amputations among children and adolescents globally from 1990 to 2021.

In 1990, the transnational SII for the global burden of amputation among children and adolescents revealed differences in the incidence, prevalence, and YLDs of 106, 1,221, and 4 cases per 100,000 people, respectively, between countries with the highest and lowest SDI. By 2021, these disparities had slightly increased to 101, 1,299, and 6 cases per 100,000 people, respectively (Figures 3A,C,E; Supplementary Table S5). These data indicate that the absolute inequality in the burden of amputation between high-SDI and low-SDI countries has widened over the past three decades.

Further analyses of the relative inequality revealed that the concentration index decreased by 0.09, 0.06, and 0.06, respectively, between 1990 and 2021. This decline suggests that, while high-income countries continue to bear the highest absolute burden of amputation, the relative burden has increasingly shifted toward low-income countries and regions. Such a trend underscores the significant deficits in medical resources, amputation prevention, and rehabilitation care in these regions, leading to a disproportionately higher disease burden compared to high-income countries. This growing inequality presents substantial challenges for global health policies, emphasizing the urgent need for targeted interventions to address these disparities, particularly in resource-limited regions (Figures 3B,D,F; Supplementary Table S5).

### Trends in amputations in children and adolescents from 1990 to 2040

**Figure 4:**
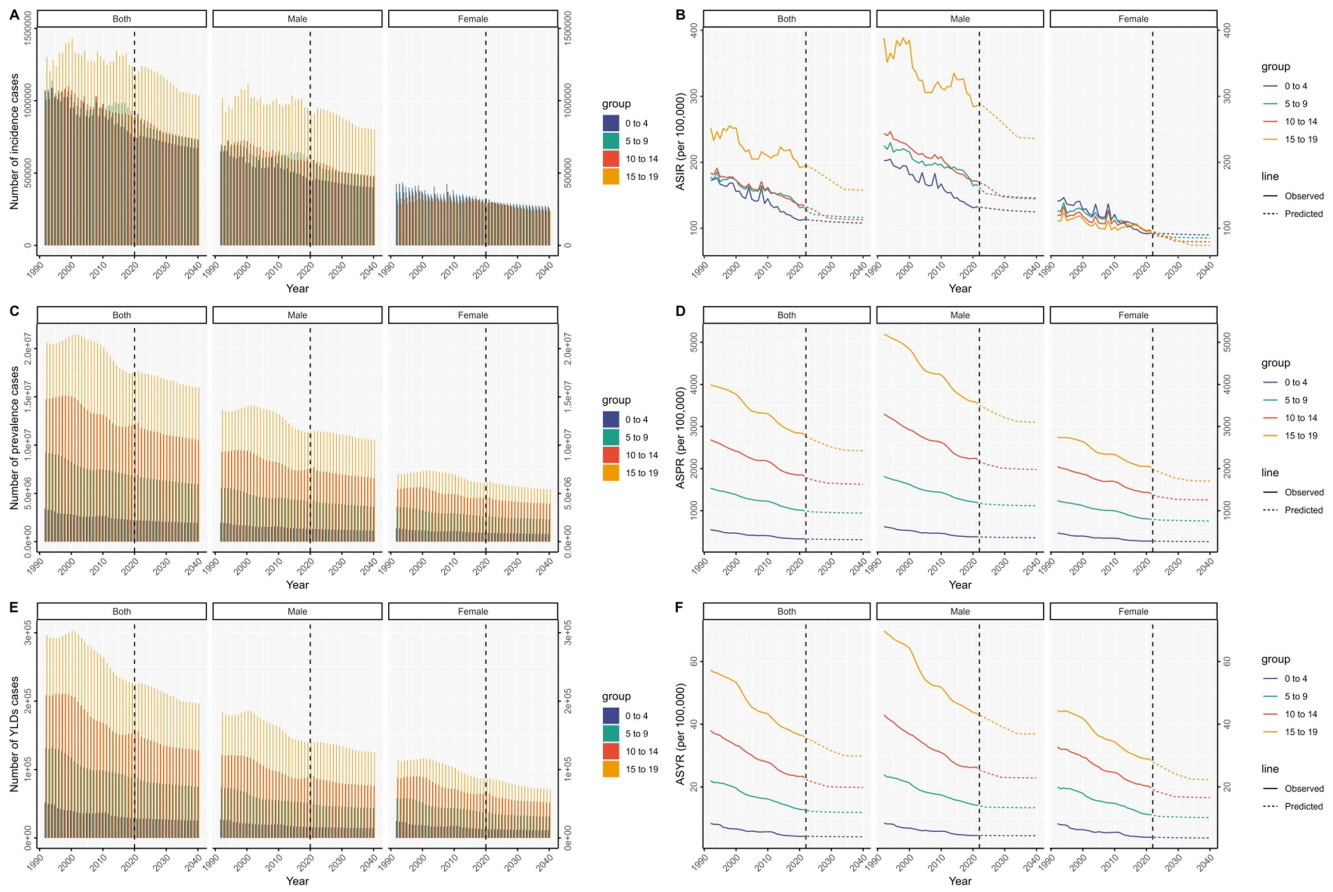
Projections of the number of incidence **(A)**, prevalence **(C)**, and YLDs **(E)** cases, and ASIR **(B)**, ASPR **(D)**, and ASYR **(F)** for males and females across different age groups globally from 1990 to 2040.

Based on future projections, the amputation burden among children and adolescents is expected to continue decreasing from 2021 to 2040 (Figure 4). Specifically, the incidence, prevalence, and YLDs for the 10–14 years and 15–19 years age groups are expected to continue showing a gradual downward trend. Meanwhile, the incidence and prevalence for the 0–4 years and 5–9 years age groups are also expected to decline, but at a slower rate. This may be due to the fact that factors influencing amputation in children over 10 years of age are more easily improved, leading to a greater reduction.

As age increases, the decline in ASIR, ASPR, and ASYR for males is expected to be more pronounced due to their higher initial incidence. In contrast, for females, the ASIR, ASPR, and ASYR are expected to remain relatively stable across age groups, showing a slower, continuous downward trend.

### Frontier analysis

**Figure 5:**
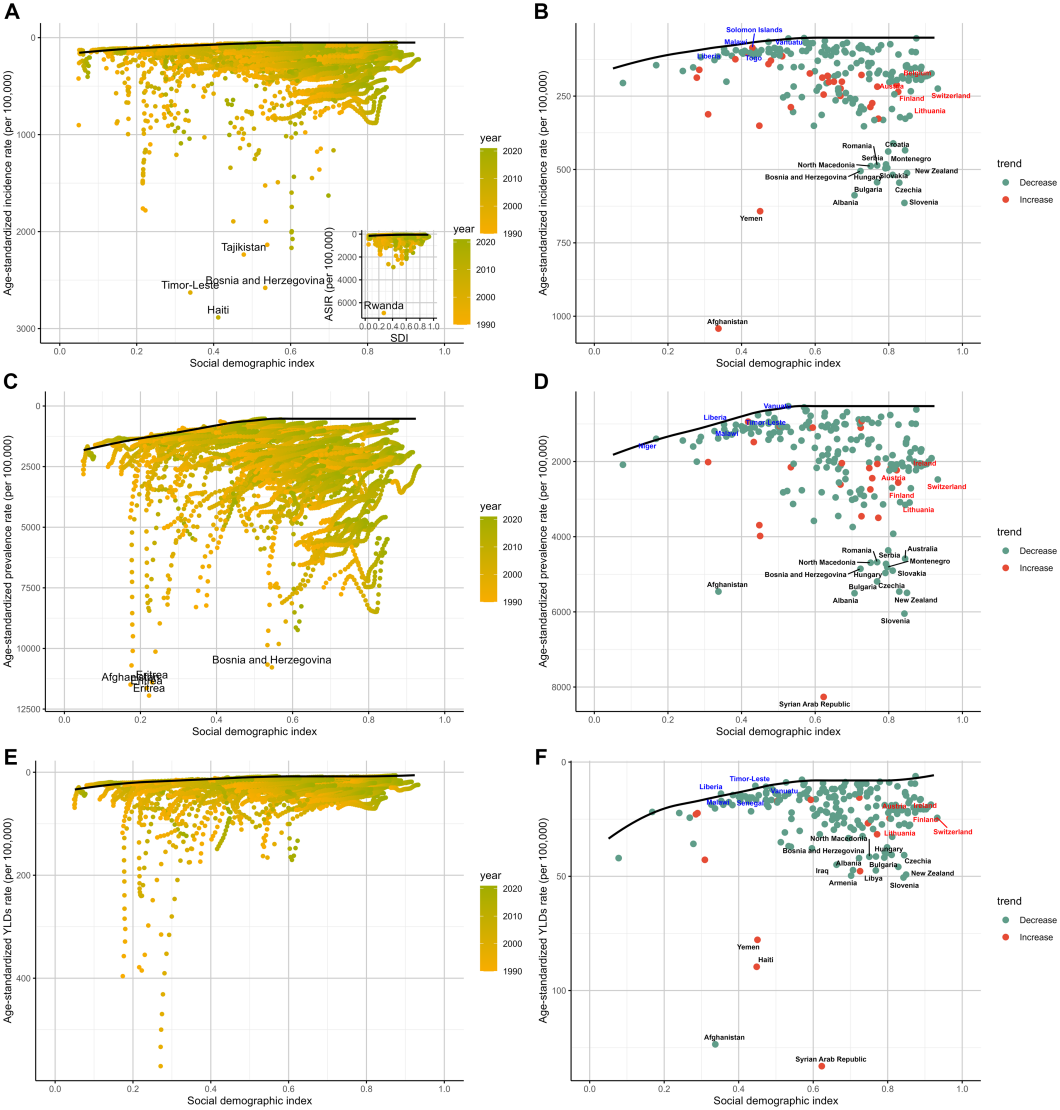
The frontier analysis, represented by a solid black line, explores the relationship between the SDI and ASR in the context of amputations among children and adolescents, covering incidence **(A,B)**, prevalence **(C,D)**, and YLDs **(E,F)**. The gradient colors in panels **(A,C,E)** indicate the change over time, with the lighter shades representing 1990 and the darkest shades representing 2021. In panels **(B,D,F)** each point represents a specific country or region in 2021, with the 15 countries showing the greatest deviation from the frontier marked in black. Countries with low SDI (> 0.5) and minimal deviation from the frontier are highlighted in blue, while those with high SDI (>0.85) and significant deviation in development levels are highlighted in red. The direction of ASR change from 1990 to 2021 is indicated by the color of the dots, with decreasing dots showing a decline and increasing dots indicating a rise.

A comprehensive frontier analysis of the SDI and ASRs for amputations among children and adolescents across 204 countries and territories from 1990 to 2021 reveals clear trends. Regarding the incidence, as the SDI values increased from 0.0 to 1.0, the ASR for amputations generally decreased. This is characterized by a gradient from lighter to darker shades over time, indicating an overall decline in prevalence. Similarly, as SDI increased, the prevalence rate of amputations decreased, and the YLDs followed a similar pattern, suggesting that the burden of amputations among children and adolescents tended to decrease with economic development (Figure 5; Supplementary Table S6).

Focusing on the 2021 frontier analysis results, the visualization illustrates the distinct disparities between countries and regions. In terms of incidence, 15 countries, including Afghanistan, Yemen, and Slovenia, exhibited significantly higher rates, placing them far from the frontier. In contrast, countries such as Liberia, Malawi, Togo, and Vanuatu are closer to the frontier, indicating optimal outcomes given their SDI levels. When assessing prevalence, Afghanistan, the Syrian Arab Republic, New Zealand, and Slovenia displayed greater deviations from the frontier. Interestingly, high-SDI countries like Austria, Ireland, and Lithuania showed relatively higher effective discrepancies at their level of development. Lastly, in the YLDs analysis, countries such as Liberia, Timor-Leste, and Malawi exhibit ratios closer to the ideal benchmark set by the frontier (Figure 5).

## Discussion

Based on an analysis of the GBD database, this study provides a detailed exploration of global epidemiological trends in amputations among children and adolescents aged 0–19 years between 1990 and 2021. The results indicate that the incidence, prevalence, and YLDs of amputation in this age group have declined globally over the past 30 years, particularly in countries and regions with a high SDI. However, significant regional and sex disparities in the disease burden persist, and the underlying causes require further investigation.

It should be noted that although the GBD database provides global data, the data collection capabilities and healthcare infrastructure in low-income countries and regions are often insufficient, which may affect the completeness and accuracy of the data. For example, medical records in these regions may be incomplete, and there are limitations in data collection and processing capabilities, which could result in data from some regions not fully reflecting the true amputation burden. Additionally, global healthcare resources and health interventions are unevenly distributed, and the methods of health data collection and processing in low-income regions may differ significantly from those in high-income regions. Therefore, caution is required when using these data, considering the potential biases.

Regarding the focus of this study on the 0–19 age group, the primary reason for choosing this age range is that the impact of amputation on children and adolescents is particularly profound, and the physical, psychological, and social consequences faced by this group are often more significant than those in adults. For adolescents, amputation is not only a major physical loss but also has a far-reaching impact on their mental health, social integration, and future quality of life. Unlike adult populations, adolescents face additional challenges in education and employment, especially in high-risk areas, making the needs of this group more urgent. Therefore, focusing on the amputation burden in the 0–19 age group provides more specific information for policymakers and public health interventions, helping to develop more targeted measures.

While we acknowledge that focusing solely on the 0–19 age group may not fully reflect global amputation trends, this research design allows us to delve deeper into the amputation impact on children and adolescents, filling a gap in the literature. It provides more precise data to support global health policy improvement and resource allocation.

First, the overall decline in the global burden of amputations reflects advancements in medical technology, especially rehabilitation in trauma management, surgical techniques, and postamputation (28). High-income countries have notable advantages in trauma treatment, prosthetic technology, and rehabilitation services, leading to better outcomes for patients postamputation. This trend is particularly evident in high-SDI countries, where, despite higher incidence and prevalence rates, YLDs remain low, suggesting that efficient healthcare systems play a crucial role in minimizing disability burdens. In contrast, low-SDI countries often experience poor outcomes due to inadequate medical resources and treatment capacities, resulting in higher YLD rates.

Second, this study highlights substantial sex and age differences in the global burden of amputations. The incidence, prevalence, and YLD rates were higher among male children and adolescents than among females, particularly in the 15–19 years age group. This disparity may be attributed to the fact that male adolescents are more likely to engage in high-risk activities, physical labor, and exposure to hazardous environments (29, 30). Sociocultural factors, including gender expectations and societal norms, might also contribute to these differences. For example, in many societies, males are often expected to take part in more physically demanding and dangerous activities, such as manual labor or high-risk sports. These social and cultural pressures may make adolescent males more vulnerable to injuries leading to amputation. Furthermore, as boys age, the gap between males and females in terms of amputation risk becomes more pronounced. This suggests that puberty and the transition to adulthood might further expose young males to risks that increase their likelihood of amputation. Therefore, public health policies aimed at reducing amputation risks in males, especially in the 15–19 age group, should focus on promoting safety in high-risk activities and educating young males about the importance of protective measures. In contrast, females, particularly in the same age group, appear to have a lower risk of amputation. This could be related to lower levels of engagement in high-risk activities or a more cautious approach to physical labor and risky behaviors. However, this gender difference might also reflect broader societal and cultural factors, where girls may be less likely to engage in activities that could expose them to higher risks of injury. Nevertheless, further research is needed to fully understand these gender differences and their implications for targeted public health interventions.

Age distribution analyses revealed that the incidence, prevalence, and YLD rates increased with age, with the highest burden occurring among adolescents aged 15–19 years. Adolescents in this age range are at a higher risk of exposure to trauma, traffic accidents, and physically demanding work, especially in low-income countries where young people are more likely to engage in high-risk activities, directly contributing to higher amputation rates. Additionally, the early onset of diabetes has become more common. A US study reported a 4.8% annual increase in the incidence of type 2 diabetes among 10–19-year-olds from 2002–2003 to 2011–2012 (31). While diabetes is a major cause of amputation in adults (32), its role in amputations among adolescents remains less clear. However, the increasing prevalence of type 2 diabetes among young people may contribute to an elevated risk of amputation in the future.

In high-SDI countries, the decline in amputation rates has been significantly influenced by advancements in medical technology, including trauma care, surgical techniques, and rehabilitation services. These countries have better access to trauma treatment, which helps improve outcomes for amputees. Additionally, improvements in rehabilitation services in high-SDI countries play a crucial role in minimizing the long-term impact of amputation. For example, physical therapy and psychological support help significantly improve the quality of life for amputees and reduce the burden of YLDs.

In contrast, in low-SDI regions and conflict-affected countries, the slow decline in amputation rates presents challenges due to inadequate medical resources and limited access to rehabilitation services. These regions often lack comprehensive trauma care systems, and amputees in these areas face difficulties in receiving proper rehabilitation, leading to higher YLD rates. The lack of medical infrastructure, combined with limited access to prosthetic care, exacerbates the disability burden in low-SDI countries, where amputees may not receive the same level of post-surgical rehabilitation and prosthetic support as those in high-SDI regions.

While the overall trend in the global burden of amputation is decreasing, the rate of change varies significantly by region. For example, East Sub-Saharan Africa has experienced the most significant decline, reflecting substantial improvements in trauma care and healthcare infrastructure. However, other regions, such as the Caribbean, have seen an increase in incidence and prevalence, which may be associated with frequent natural disasters (33). The potential link between suicides and amputations is primarily related to failed suicide attempts, where survivors may suffer severe injuries that necessitate amputation. There is also a continued rise in youth suicide rates in the Caribbean, now the third leading cause of death for those aged 10–19 years (34), which may be a contributing factor. Future public health strategies in these high-burden areas should not only focus on enhancing trauma treatment and rehabilitation services but also address adolescent mental health, especially in low-income countries with high trauma rates.

Additionally, war and social conflict remain significant drivers of the burden of amputations in certain regions. Studies have shown that countries such as Afghanistan and Syria have significantly higher incidence and prevalence rates of amputation, correlating with prolonged conflict (35). The trauma of war not only increases the risk of amputation but also disrupts healthcare systems, preventing timely and effective treatment and rehabilitation services for injured children and adolescents. This underscores the profound impact of social environments on health burdens. In the future, international efforts should focus on providing medical assistance to war-torn regions, especially in trauma treatment and post-conflict rehabilitation, to mitigate the long-term health impacts on children and adolescents.

Finally, future projections suggest that while the global burden of amputations among children and adolescents will continue to decrease through 2040, certain high-risk regions such as Afghanistan and Syria may experience persistent or even increasing burdens. This indicates that despite overall global improvements, these high-risk areas require special attention. Public health policies and international aid should prioritize enhancing medical care in these regions, particularly focusing on trauma prevention and rehabilitation services to further reduce the amputation burden (36). Although the Nordpred model provides a valuable perspective for forecasting the burden of amputations, it has certain limitations. First, the model assumes that historical trends will continue unchanged. However, in reality, changes in socioeconomic conditions, advancements in medical technology, and shifts in public health policies may significantly affect the future burden of amputations. As a result, the model’s predictions may not fully reflect future realities, especially in the context of sudden global health events or rapid technological developments. Secondly, while the linear extrapolation assumption of the Nordpred model is applicable to many trends, it may introduce considerable error when facing complex or nonlinear changes. The accuracy of predictions may be compromised, particularly in scenarios involving significant innovations in medical technology or major policy reforms. Thirdly, the model relies primarily on historical data, and disparities in healthcare infrastructure and data collection capacity—especially in low- and middle-income countries—may affect the completeness and reliability of the input data, thereby limiting the robustness of the predictions. Lastly, apart from the Joinpoint model which fully accounted for UIs, other models in this study did not incorporate UI information. This represents a limitation of our current work. We hope to identify effective methods to address this issue in future research.

International cooperation plays a crucial role in improving healthcare conditions and rehabilitation services in conflict-affected countries. Countries affected by war, such as Afghanistan and Syria, often face severe destruction of healthcare infrastructure and a lack of resources, making timely treatment and effective rehabilitation challenging. The international community can support these countries by providing essential medical resources, training, and infrastructure development through humanitarian aid and cooperation. International organizations and NGOs (such as the World Health Organization, Red Cross, etc.) can deploy medical teams, equipment, and supplies to help improve the treatment outcomes for injured children and adolescents. Additionally, establishing long-term rehabilitation support systems is crucial to assist injured adolescents in physical, psychological, and social recovery.

For low-SDI regions, resource allocation and policy interventions are equally important. Low- and middle-income countries often lack adequate medical resources and effective prevention measures, which results in a heavier burden of amputation. Policy interventions should prioritize improving the healthcare infrastructure and rehabilitation services in these regions. For instance, through international funding and public health policy support, investments in hospitals and trauma treatment centers in low-SDI areas can be enhanced, while improving the skills of local healthcare workers to ensure timely and effective treatment for trauma patients. Raising awareness and providing specialized rehabilitation services for amputees, such as prosthetics and follow-up care, could significantly improve their quality of life and reduce disability burdens due to inadequate healthcare resources.

Furthermore, policy interventions should be tailored to specific regional characteristics and take more targeted approaches. For example, in low-income countries and conflict-affected regions, focusing on traffic safety, occupational injury prevention, and public education would help reduce amputation rates by minimizing exposure to high-risk activities, raising public safety awareness, and promoting the use of protective equipment. For high-risk populations, such as male adolescents and those engaged in dangerous jobs, targeted interventions should be implemented, such as strengthening legal protections for adolescent labor in high-risk environments to reduce the incidence of traumatic injuries leading to amputations.

Future research could provide more feasible policy recommendations through case studies to guide public health practice. Specifically, future studies could focus on representative low-SDI countries or conflict-affected regions, assessing existing public health policies and collecting data on their impact. This would help identify which policies are effective, which strategies need improvement, and offer practical insights for international organizations, governments, and public health practitioners. By evaluating real-world examples, future research can further advance global health policy and offer targeted solutions for reducing the burden of amputations.

## Conclusion

This study reveals a consistent downward trend in amputations among children and adolescents worldwide, particularly in high-income countries, where advances in medical technology and improved trauma care have been instrumental. However, the burden remains high in low-income countries and conflict-prone regions, necessitating future public health strategies that emphasize resource allocation and trauma prevention. Mental health prevention for adolescents, particularly males, should also be strengthened to reduce amputation rates. With these interventions, the global burden of amputations in children and adolescents can be further reduced, leading to better long-term health outcomes.

## Data Availability

The original contributions presented in the study are included in the article/Supplementary material, further inquiries can be directed to the corresponding authors.

## Supplementary material

SUPPLEMENTARY FIGURE S1 Number of incidence, prevalence, and YLDs of amputations across different age groups by SDI quintiles from 1990 to 2021.

SUPPLEMENTARY FIGURE S2 Trends in the age-standardized rates (ASR) of amputations across different age groups and SDI regions globally.

SUPPLEMENTARY FIGURE S3 Trends in age-standardized rates (ASR) of amputations among children and adolescents across global GBD regions from 1990 to 2021.

SUPPLEMENTARY FIGURE S4 Number of incidence, prevalence, and YLDs of amputations among children and adolescents by sex across GBD regions from 1990 to 2021.

SUPPLEMENTARY FIGURE S5 Comparison of trends in the incidence, prevalence, YLDs, and ASR of amputations among children and adolescents globally and across five SDI regions by sex and age groups in 1990 and 2021. **(A)** represents global data, **(B)** high SDI, **(C)** high–middle SDI, **(D)** middle SDI, **(E)** low–middle SDI, and **(F)** low SDI.

SUPPLEMENTARY FIGURE S6 Average annual percentage change (AAPC) of ASIR, ASPR, and ASYR by GBD region and sex from 1990 to 2021.

SUPPLEMENTARY FIGURE S7 AAPC trends of age-standardized rates (ASR) for amputations across SDI regions (1990–2021). The panels illustrate the annual percentage changes (APC) in incidence **(A)**, prevalence **(B)**, and YLDs **(C)** for amputations among children and adolescents across various SDI regions globally. Each line represents ASR trends for the overall age groups within different SDI categories (high, high–middle, middle, low–middle, and low SDI).

SUPPLEMENTARY FIGURE S8 AAPC trends of age-standardized rates (ASR) of amputations in SDI regions (1990–2021). The panels illustrate the annual percentage change (APC) of incidence **(A)**, prevalence **(B)**, and YLDs **(C)** among adolescents aged 10–19 across different SDI regions globally. Each line represents the ASR trend for overall rates within various SDI categories (high, high–middle, middle, low–middle, and low SDI), highlighting differences in trends across these socio-demographic index regions.

SUPPLEMENTARY FIGURE S9 AAPC trends of age-standardized rates (ASR) of amputations in SDI regions (1990–2021). The panels illustrate the annual percentage change (APC) of the incidence **(A)**, prevalence **(B)**, and YLDs **(C)** among adolescents aged 0–9 years across different SDI regions globally. Each line represents the ASR trend for overall rates within various SDI categories (high, high-middle, middle, low–middle, and low SDI), highlighting differences in trends across these socio-demographic index regions.

SUPPLEMENTARY FIGURE S10 Geographical distribution of amputation incidence among children and adolescents across 204 countries and regions. **(A)** The age-standardized incidence rate (ASIR) of amputations in children and adolescents in 2021. **(B)** The relative change in incidence counts for children and adolescents from 1990 to 2021 across different regions. **(C)** The average annual percentage change (AAPC) of ASIR for amputations in children and adolescents from 1990 to 2021 across various regions.

SUPPLEMENTARY FIGURE S11 Geographical distribution of amputation prevalence among children and adolescents across 204 countries and regions. **(A)** The age-standardized prevalence rate (ASPR) of amputations in children and adolescents in 2021. **(B)** The relative change in prevalence counts (%) for children and adolescents from 1990 to 2021 across different regions. **(C)** The average annual percentage change (AAPC) of ASPR for amputations in children and adolescents from 1990 to 2021 across various regions.

SUPPLEMENTARY FIGURE S12 Geographical distribution of age-standardized YLD rates (ASYR) related to amputations among children and adolescents across 204 countries and regions. **(A)** The age-standardized YLD rate (ASYR) of amputations in children and adolescents in 2021. **(B)** The relative change in YLD counts (%) for children and adolescents from 1990 to 2021 across different regions. **(C)** The average annual percentage change (AAPC) of ASYR for amputations among children and adolescents from 1990 to 2021 across various regions.

SUPPLEMENTARY FIGURE S13 Projected numbers and ASR of incidence, prevalence, and YLDs for amputations among children and adolescents, by sex (both, male, and female) from 1990 to 2040.

SUPPLEMENTARY TABLE S1 The incidence of amputation among children and adolescents in 1990 and 2021 and the annual percentage change by region.

SUPPLEMENTARY TABLE S2 The prevalence of amputation among children and adolescents in 1990 and 2021 and the annual percentage change by region.

SUPPLEMENTARY TABLE S3 The YLDs of amputation among children and adolescents in 1990 and 2021 and the annual percentage change by region..

SUPPLEMENTARY TABLE S4 Age–period–cohort analysis for different genders.

SUPPLEMENTARY TABLE S5 The transnational inequality analysis of amputations among children and adolescents from 1990 to 2021.

SUPPLEMENTARY TABLE S6 Frontier of amputation incidence, prevalence, YLDs rate among children and adolescents and effective.
