# Supplementary figures and images for: Global burden of amputation among children and adolescents from 1990 to 2021: systematic analysis of the Global Burden of Disease study 2021
Source: Front Public Health. 2025 Jun 27;13:1589309. doi: 10.3389/fpubh.2025.1589309 (PMC12277607; doi:10.3389/fpubh.2025.1589309)

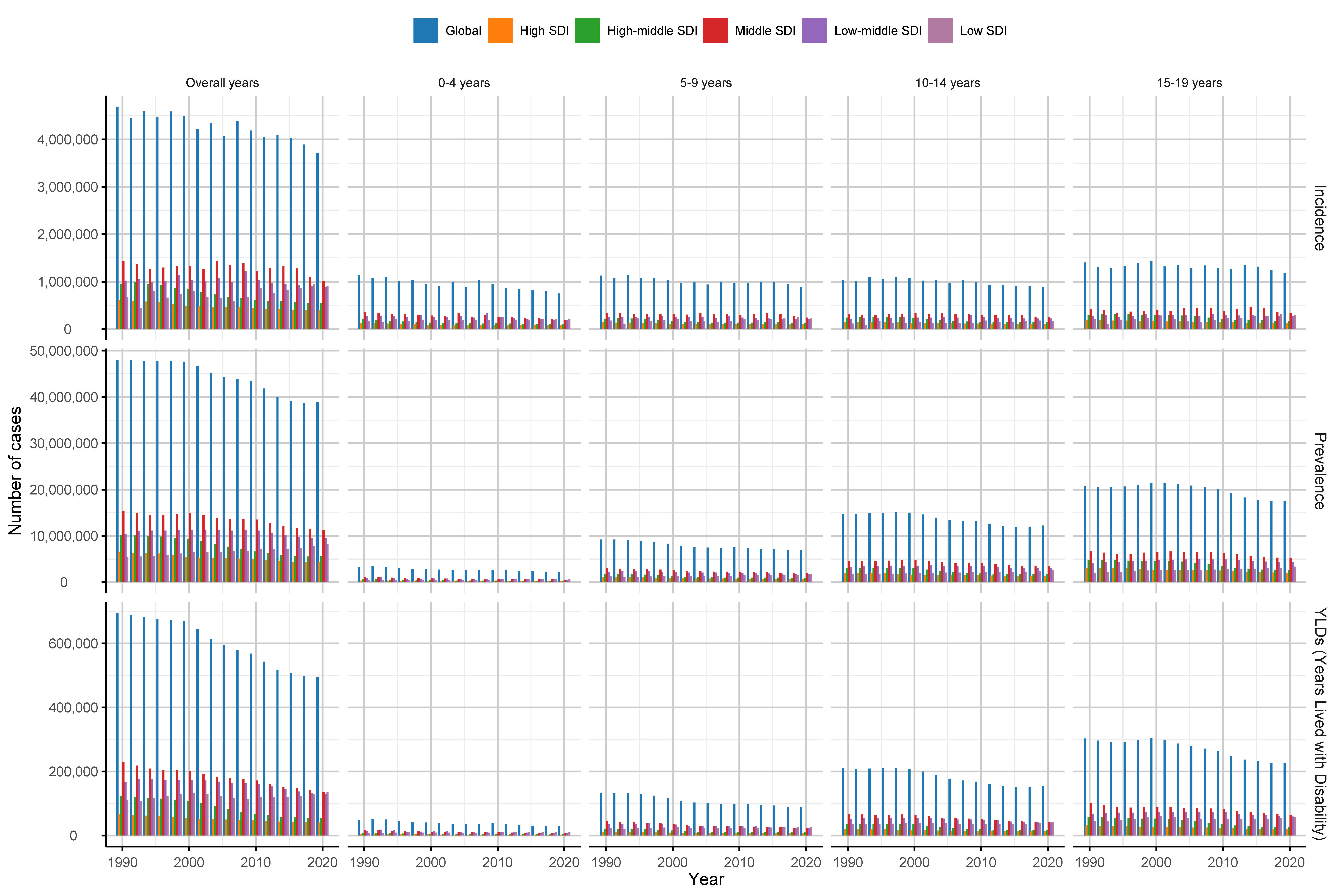

Supplement: SUPPLEMENTARY FIGURE S1 — Number of incidence, prevalence, and YLDs of amputations across different age groups by SDI quintiles from 1990 to 2021. [file Image_1.tif]

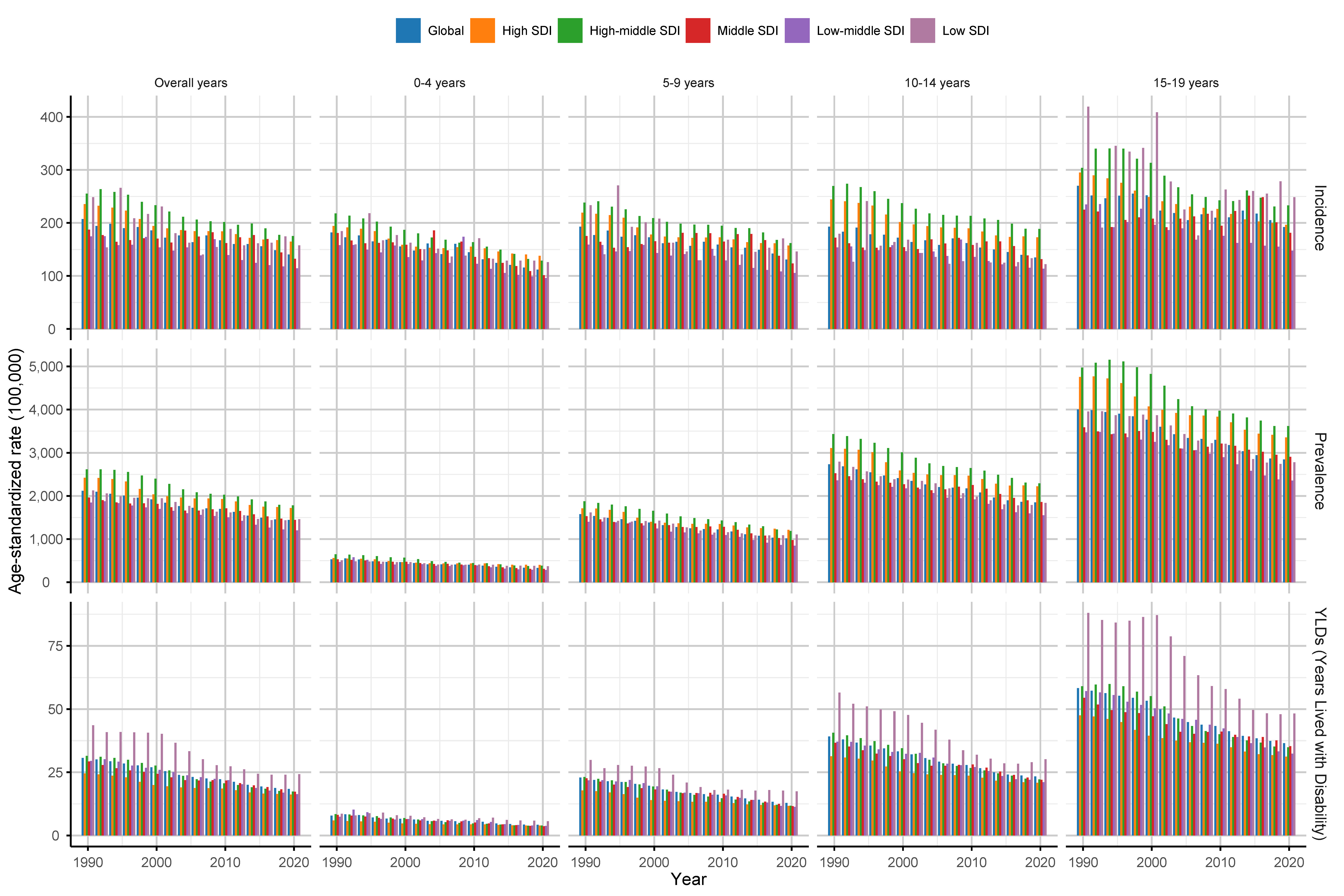

Supplement: SUPPLEMENTARY FIGURE S2 — Trends in the age-standardized rates (ASR) of amputations across different age groups and SDI regions globally. [file Image_2.tif]

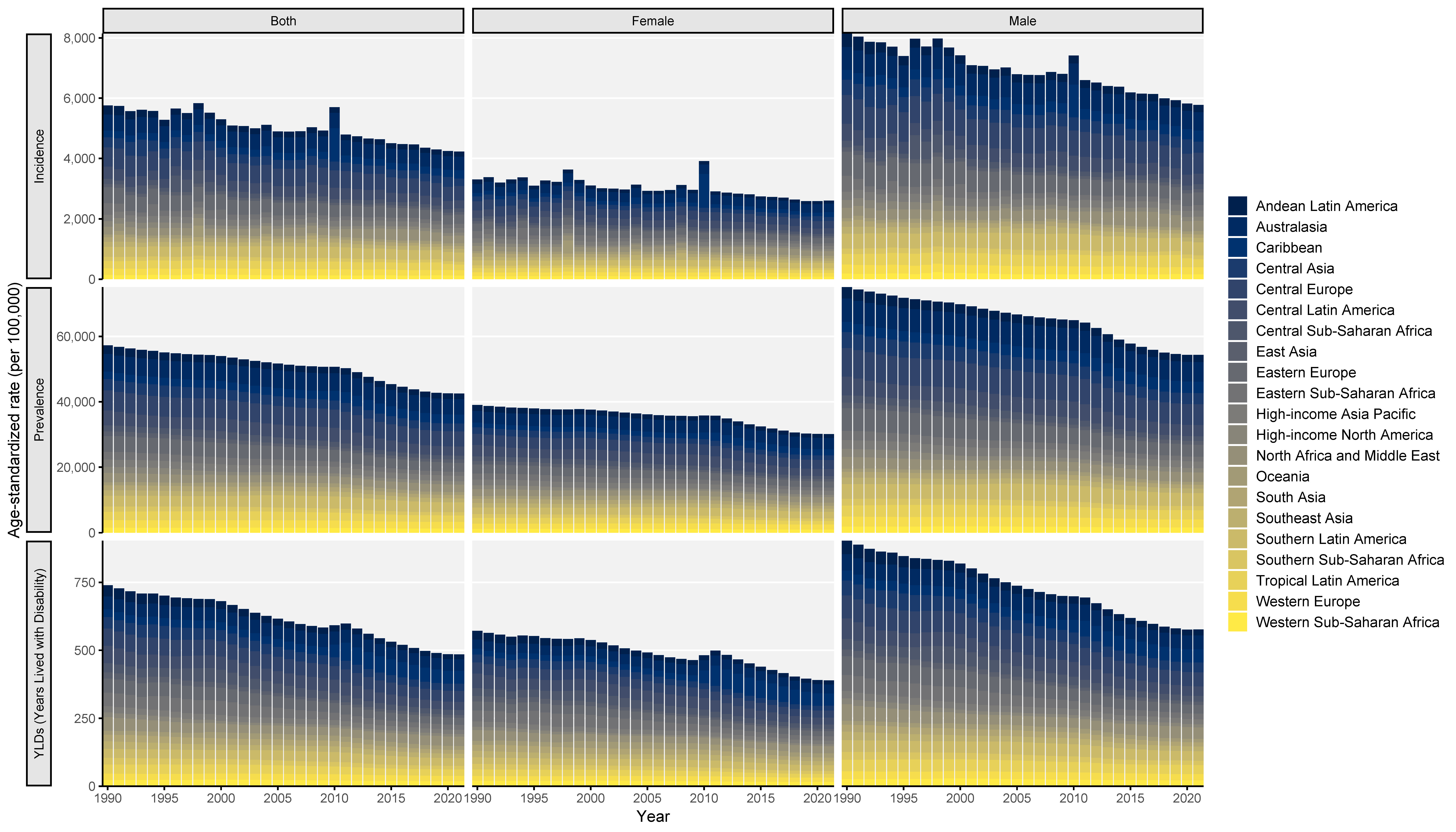

Supplement: SUPPLEMENTARY FIGURE S3 — Trends in age-standardized rates (ASR) of amputations among children and adolescents across global GBD regions from 1990 to 2021. [file Image_3.tif]

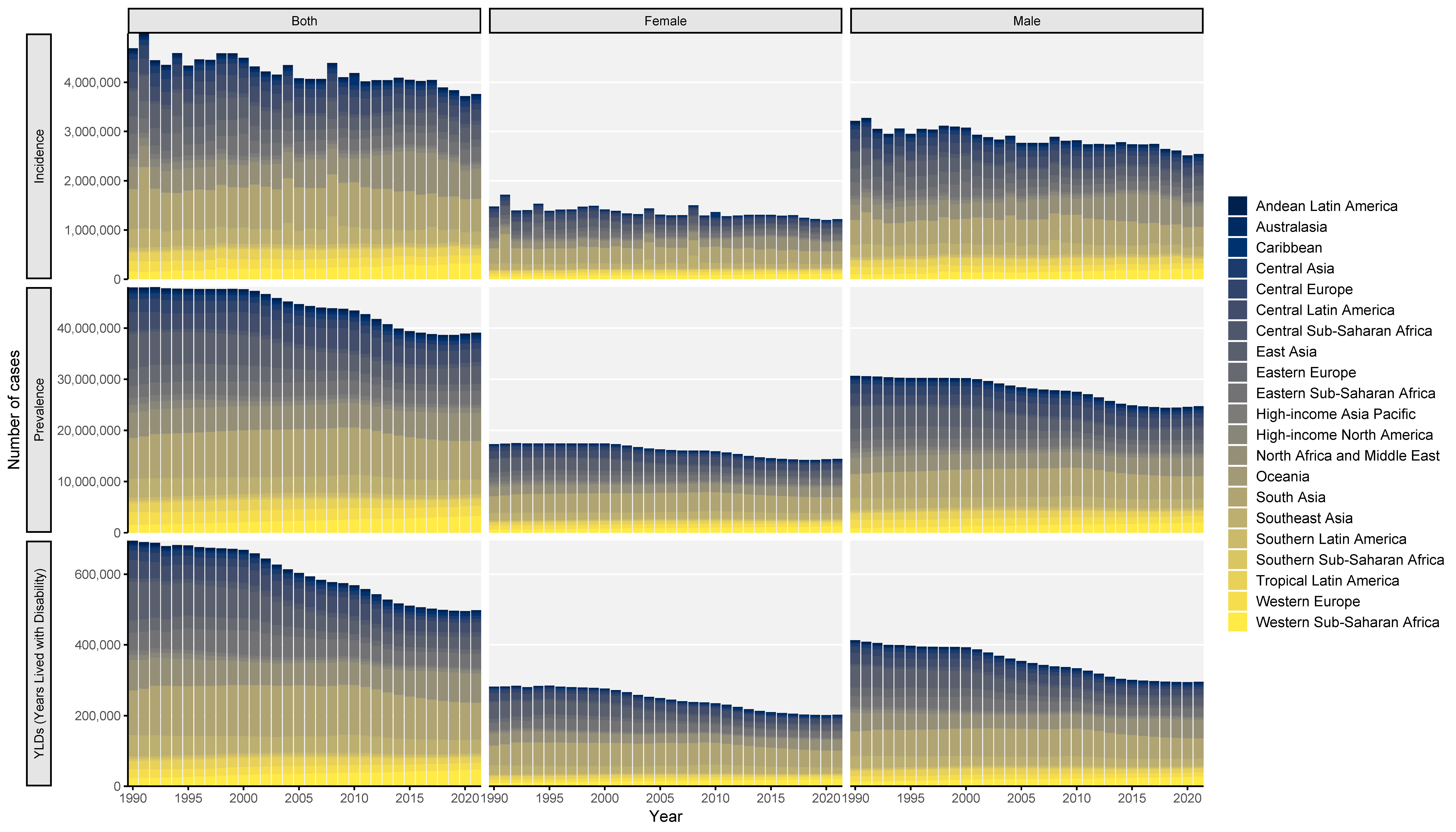

Supplement: SUPPLEMENTARY FIGURE S4 — Number of incidence, prevalence, and YLDs of amputations among children and adolescents by sex across GBD regions from 1990 to 2021. [file Image_4.tiff]

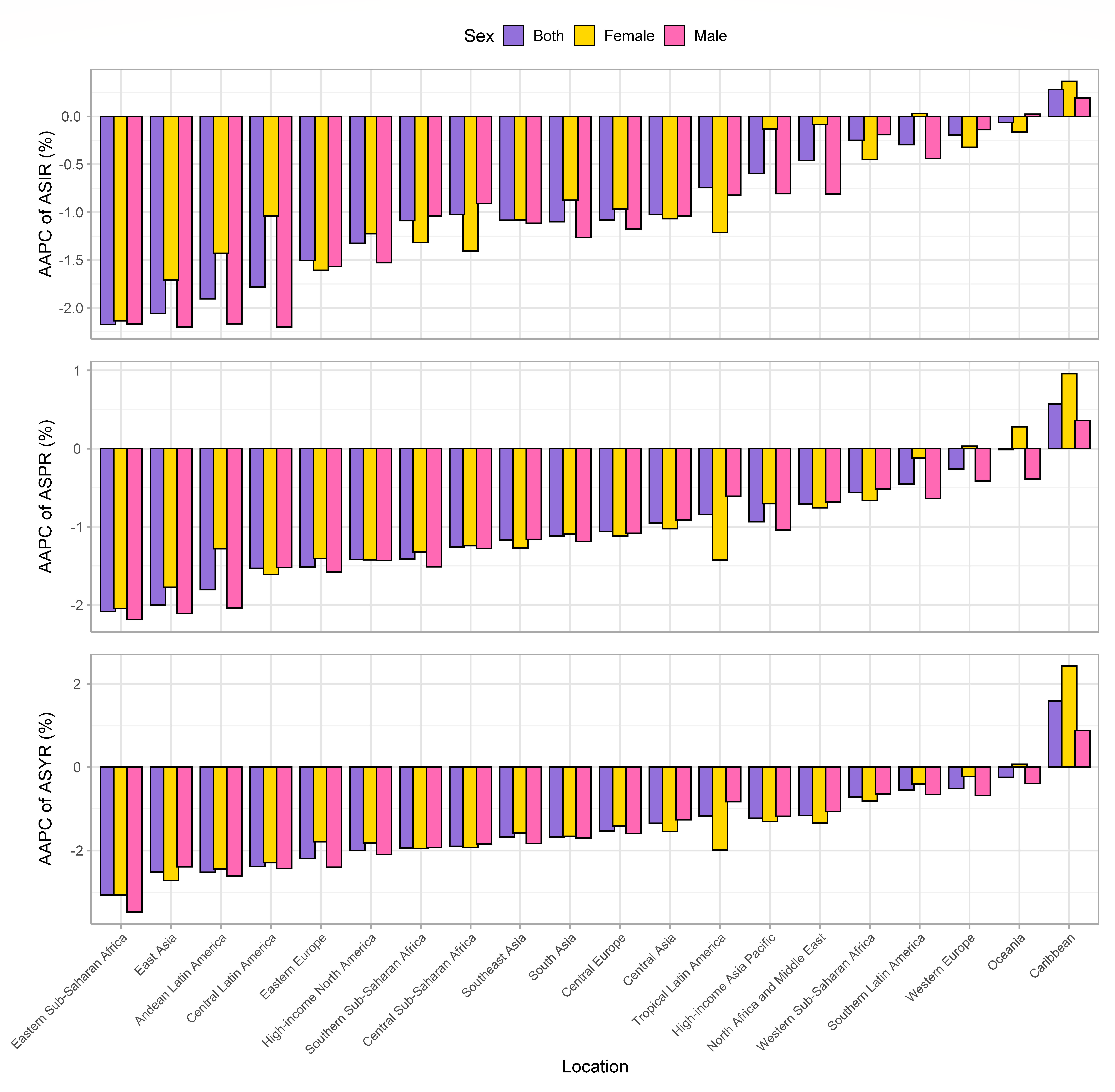

Supplement: SUPPLEMENTARY FIGURE S6 — Average annual percentage change (AAPC) of ASIR, ASPR, and ASYR by GBD region and sex from 1990 to 2021. [file Image_6.tiff]

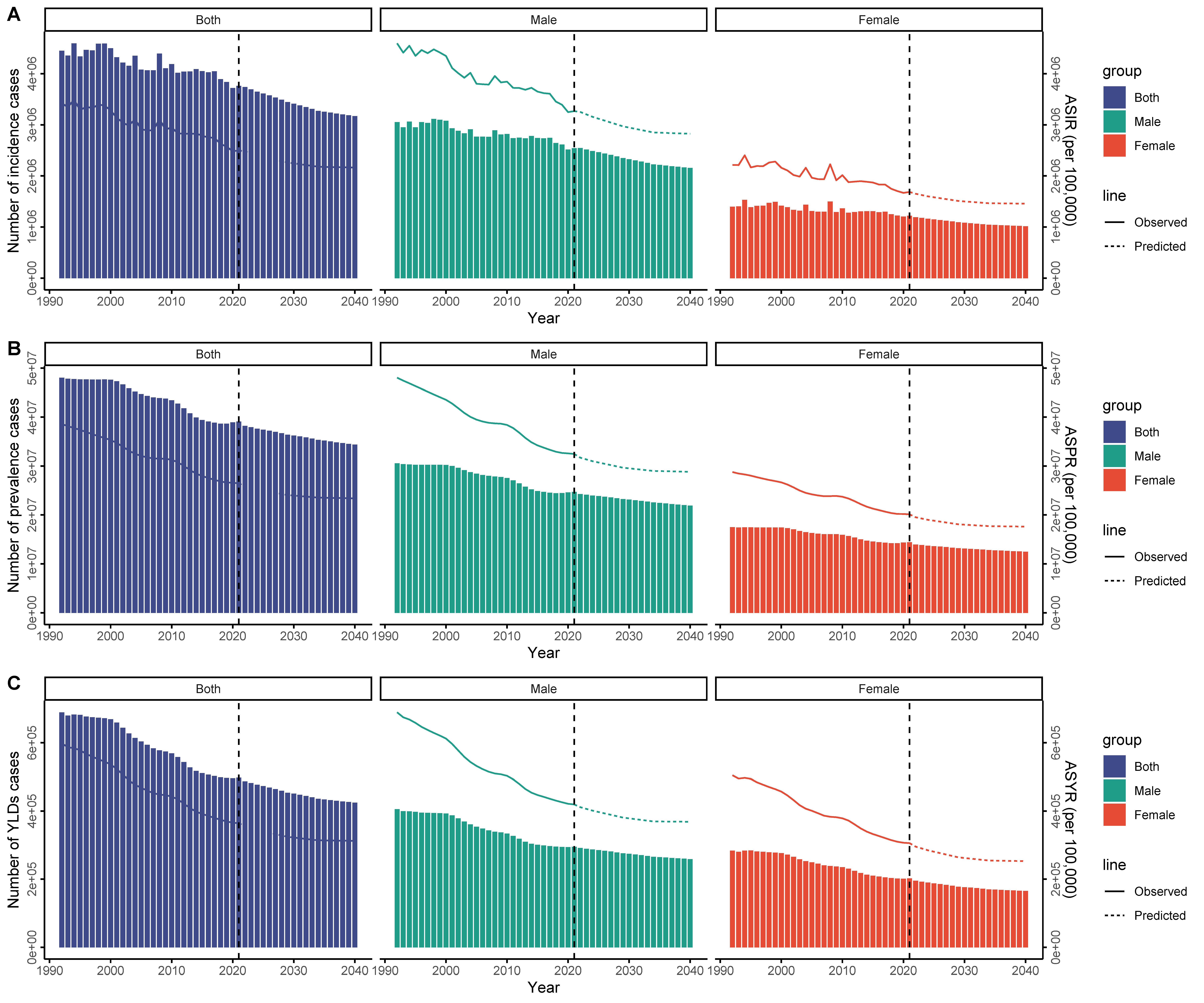

Supplement: SUPPLEMENTARY FIGURE S13 — Projected numbers and ASR of incidence, prevalence, and YLDs for amputations among children and adolescents, by sex (both, male, and female) from 1990 to 2040. [file Image_13.tiff]
